# Experimental realization of a multiplexed quantum memory with 225 individually accessible memory cells

**DOI:** 10.1038/ncomms15359

**Published:** 2017-05-08

**Authors:** Y-F Pu, N. Jiang, W. Chang, H-X Yang, C. Li, L-M Duan

**Affiliations:** 1 Center for Quantum Information, IIIS, Tsinghua University, Beijing 100084, China; 2 Department of Physics, University of Michigan, Ann Arbor, Michigan 48109, USA

## Abstract

To realize long-distance quantum communication and quantum network, it is required to have multiplexed quantum memory with many memory cells. Each memory cell needs to be individually addressable and independently accessible. Here we report an experiment that realizes a multiplexed DLCZ-type quantum memory with 225 individually accessible memory cells in a macroscopic atomic ensemble. As a key element for quantum repeaters, we demonstrate that entanglement with flying optical qubits can be stored into any neighboring memory cells and read out after a programmable time with high fidelity. Experimental realization of a multiplexed quantum memory with many individually accessible memory cells and programmable control of its addressing and readout makes an important step for its application in quantum information technology.

## Abstract (web summary)

To realize long-distance quantum communication it is required to have individually addressable quantum memories with programmable access to many cells. Here the authors report DLCZ-type quantum memories with 225 individually accessible memory cells in a macroscopic atomic ensemble

For realization of long-distance quantum communication through the quantum repeater network^1^,^2^,^3^,^4^,^5^,^6^ or linear optics quantum computation using atomic ensembles^7^,^8^, a key requirement is to have multiplexed quantum memory with many memory cells. Each memory cell needs to be individually accessible with access time independent of physical location of the cells. A well-known implementation of the quantum repeater network is through the Duan-Lukin-Cirac-Zoller (DLCZ) scheme^2^, where probabilistically generated atom-photon entanglement is stored in the DLCZ-type memory^2^,^3^,^4^,^5^. On-demand readout of the information or entanglement stored in the DLCZ memory at a programable time is critical for archiving efficient scaling of the quantum repeater scheme^1^,^2^,^3^,^5^.

Atomic internal states in the ground-state manifold provide an ideal candidate for realization of quantum memory because of their long coherence time and the possibility of an efficient interface with the quantum bus carried by the flying photonic qubits^3^,^4^. In particular, the collective internal states of a many-atom ensemble have enhanced coupling to directional light and thus provides a quantum memory with efficient interface to photons even in free space^2^,^3^,^4^,^5^. Such an ensemble-based quantum memory has found many applications^3^,^4^,^5^, in particular for realization of quantum internet and long-distance quantum communication^1^,^2^,^3^,^4^,^5^,^6^. Quantum memory with efficient interface to light has been demonstrated up to a few qubits using atomic ensembles in either vacuum or low-temperature crystals^9^,^10^,^11^,^12^,^13^,^14^,^15^,^16^,^17^,^18^,^19^,^20^. Through access to the orbital angular momentum or spatial imaging space of the photon^21^,^22^, high-dimensional photon–photon entanglement can be generated and up to seven-dimensional entanglement has been stored in the atomic ensemble^23^. To increase the memory capacity, a more efficient method is to use the multiplexed memory of multiple qubits as an *n*-qubit memory has an effective Hilbert space dimension of 2^*n*^. One can use multiplexing in the time-bin^24^,^25^,^26^, the spatial profile^15^, or the angular directions^27^ to increase the memory capacity. Multiple time-bin qubits have been mapped into and out of a solid-state atomic ensemble at pre-determined time^25^,^26^. The read-out for this system, however, is not on demand and we have only sequential instead of programmable random access to the stored information. For the multiplexing based on spatial profile or the angular directions, either the memory capacity is still limited^15^ or one has yet to achieve full quantum-limited operation^27^. To scale up the capability of quantum memory, which is critical for its application in quantum information technology^3^,^4^,^5^, we need extendable quantum systems with many memory cells and programmable access to each cell with location-independent access time similar to the classical random access memory.

In this paper, we demonstrate a multiplexed quantum memory with 225 individually accessible memory cells. This is achieved by dividing a macroscopic atomic ensemble into a two-dimensional (2D) array of micro-ensembles, each serving as a quantum memory cell. We use crossed acoustic optical deflectors (AODs) to realize a 2D multiplexing and demultiplexing optical circuit so that each memory cell can be individually addressed by the write and read laser beams and the quantum signals from each cell can be coupled into single mode fibers in a programmable way. AODs provide a convenient device for design of multiplexing circuits, which have been used recently for individual addressing of single atoms^28^, ions^29^ and one-dimensional (1D) array of ensembles^15^. The development from 1D array^15^ to the 2D geometry of multiplexing is critical for the dramatic increase of the memory capacity. To verify functionality of our multiplexed quantum memory, we perform write and read operations on a 15 × 15 2D array of memory cells, and demonstrate their quantum correlations and individual controllability with negligible crosstalk. In the DLCZ scheme, we typically use a pair of neighboring memory cells to store entanglement with flying optical qubits^2^,^3^. Through quantum state tomography, we demonstrate that entanglement with flying optical qubits can be stored into any chosen pair of neighboring memory cells and read out after a programmable time with about 90% fidelity. This constitutes an important step for realization of quantum repeaters.

## Results

### Experimental configuration

**Figure 1:**
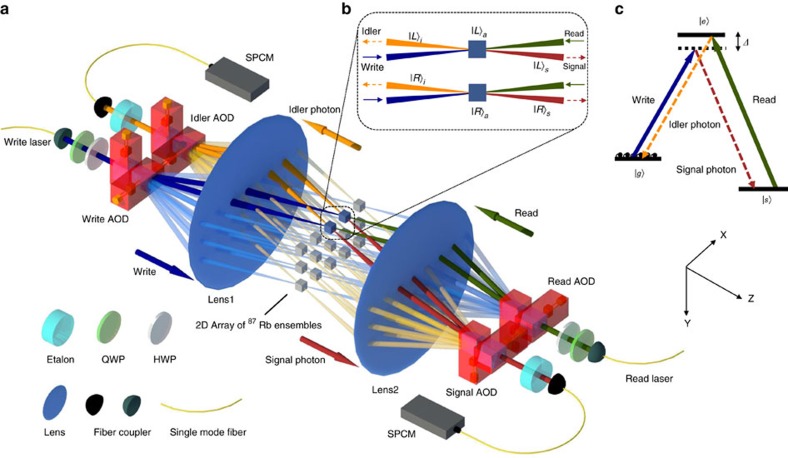
Experimental setup for demonstration of a multiplexed quantum memory with 225 memory cells. (**a**) The write and the read laser beams are directed by the multiplexing AODs to address 15 × 15 atomic memory cells contained in single macroscopic atomic ensemble, with the distance between neighboring cells of 126 μ*m*. For clarity, only 3 × 5 cells are shown in the figure. Write-in/read-out of quantum information to/from the memory cell is achieved through the DLCZ scheme. The signal and the idler photons emitted by the memory cells from different paths are combined by the de-multiplexing AODs into the same single-mode fiber for detection with a single-photon counting module (SPCM). The polarization of the write/read beam from a single mode fiber is adjusted by a quarter-wave plate (QWP) and a half-wave plate (HWP) to generate the highest first-order diffraction efficiency from the AOD. For each beam, a pair of crossed AODs in orthogonal directions *X* and *Y* are used to scan the angle of deflected beam in the corresponding directions. The lens are used to map different angles of the deflected beams to different positions at the atomic ensemble as well as to focus the beams. A Fabry-Perot cavity (etalon) is inserted in the path of the signal/idler mode to further filter our the strong write/read beam by frequency selection. (**b**) Zoom-in of the beam configuration at two memory cells denoted as L and R. (**c**) The relevant level diagram of ^87^Rb atoms for the write and read process, with 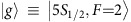, 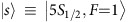, and 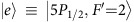. The detuning of the write beam is at Δ=10 MHz.

We use the two hyperfine states 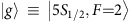 and 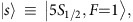 of ^87^Rb atoms to carry quantum information for the memory. Our experimental setup is shown in Fig. 1, with details described in the Methods. All the atoms are initially prepared to the state 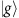 through a repumping laser pulse applied to the transition 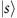
→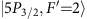. We use the DLCZ scheme to generate quantum correlation between a collective mode of the atomic ensemble and the signal photon^2^, which are propagating in the forward direction with an angle of 2° to the write laser beam. The excitation stored in the collective mode is retrieved after a controllable delay time by a read laser beam counter-propagating with the write beam, and the retrieved idler photon, counter propagating with the signal photon, is coupled into a single-mode fiber for detection.

To generate many memory cells in a single macroscopic atomic ensemble and fetch quantum signals from these cells with site-independent access time, we split the write and the read beams into 15 × 15 different paths by the 2D multiplexing optical circuit shown in Fig. 1. The corresponding signal and idler photons in each path are collected by the demultiplexing circuit on the other side. As explained in the Methods, the multiplexing and demultiplexing optical circuits are composed by crossed AODs and lens and can be programed by applying radio-frequency electric signals to direct the light to any paths or their superpositions. The phase differences between all these optical paths are intrinsically stable as they go through the same optical apparatus. We therefore do not require active phase locking between 225 different optical paths, which significantly simplifies the experimental setup.

### Characterization of quantum correlation of 225 memory cells

**Figure 2:**
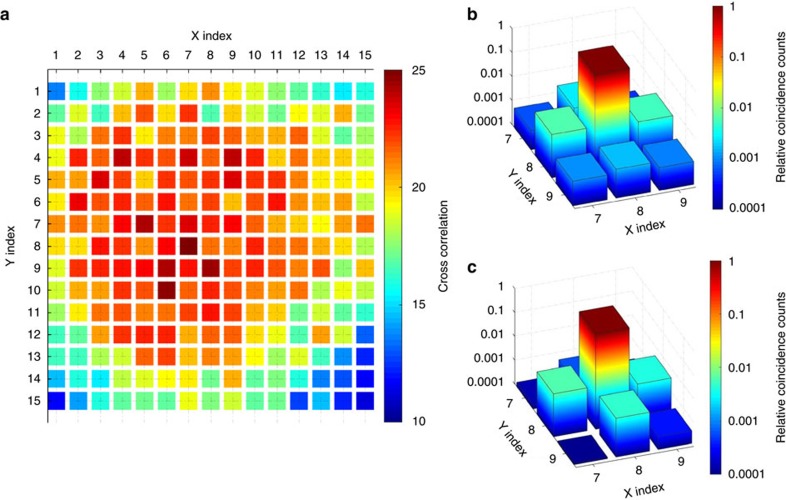
Quantum correlation and crosstalk errors in the 225-cell quantum memory. (**a**) The measured intensity cross correlation *g*_c_ between the signal and the idler photons as a function of the cell index in *X* and *Y* directions for the 2D array of 15 × 15 memory cells. The delay between the read and the write pulses is 500 ns. (**b**) The measured relative coincidence counts between the signal and the idler photons when the write beam, the signal and the idler modes are addressed to one fixed target cell (shown at the center of this figure with the cell index (8, 8)) while the read beam is scanned over the target and its neighboring cells. The relative coincidence counts, with the target one normalized to the unity, characterize the crosstalk errors of the read beams on the neighboring cells. (**c**) Similar to **b**, but with the write beam scanned over the neighboring cells, which characterize the crosstalk errors for the write beam.

To characterize quantum property of each memory cell, first we show the intensity cross-correlation *g*_c_ between the signal and the idler photons generated from each memory cell. This correlation, defined as 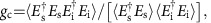 gives a signature of nonclassical property of the signal and the idler optical fields with the field operators *E*_s_ and *E*_i_ when *g*_c_>2 (ref. 30). As shown in Fig. 2a, this criterion for quantum correlation is clearly satisfied for all the 225 memory cells. When we move from the center to the edge of the ensemble, the cross correction *g*_c_ gradually decreases, which is mainly caused by the reduced optical depth from the center to the edge. The minimum *g*_c_ at the edge is still well above 10 and can be further increased if we reduce the excitation probability of the atomic mode by the write beam.

An important feature of the multi-qubit quantum memory is that different memory cells can be controlled independently without mutual influence to each other. The major crosstalk comes form the neighboring cells by the spread of the write or read laser beams. In Fig. 2b,c, we show the crosstalk errors on the neighboring sites induced by the write and the read beams. For both cases, the maximum error rate is well below 1%.

### Storage of atom-photon entanglement in memory cells

**Figure 3:**
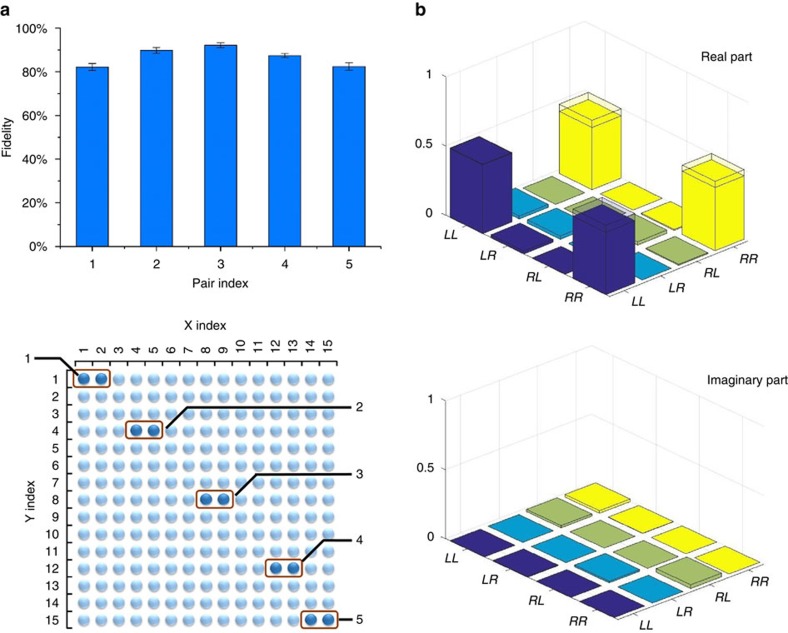
Quantum entanglement between the memory cells and the signal photon. (**a**) The measured entanglement fidelity between the chosen pairs of memory cells and the signal photon. The fidelity is calculated from the experimental density matrix reconstructed through quantum state tomography^33^. The error bars correspond to one s.d. We determine the error bars by assuming a Poissonian distribution for the photon counting statistics and propagate the error from the measured quantity to the target property (fidelity here) through numerical Monte Carlo simulation. The delay between the read and the write pulses is 500 ns. The positions of the chosen pairs of cells in the 2D memory array is shown in the lower part of the figure. (**b**) The reconstructed density matrix elements for the pair 3 of the atomic cells with the signal photon. The hollow caps denote the corresponding matrix elements for the ideal maximally entangled state.

To demonstrate storage of quantum entanglement in the memory cells, first we generate entanglement between the memory cells and the outgoing signal photons. For the DLCZ scheme, each write pulse generates with a small probability *P* a signal photon and an excitation in the corresponding collective atomic mode^2^. If the AODs equally split the write beam into two paths L and R, when an excitation is generated, it is equally distributed between the two paths, and the effective state in this case is described by


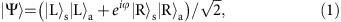


where 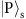 and 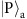 (P=L,R) denotes, respectively, a signal photon and a collective atomic excitation in the path P, and 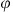 is a relative phase, which can be controlled by the electric signal applied on the AODs. Through the AODs at the demultiplexing circuit, the states 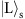 and 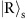 along the two paths can be combined into the output mode for detection with an arbitrary weight function. We thus can detect the signal photon in any basis 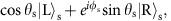 with the superposition weight function characterized by the parameters *θ*_s_ and *φ*_s_. Similarly, when we apply the read pulse on both paths L and R, the collective atomic excitation 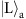 and 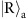, after conversion into the corresponding idler photon states, are combined by the AODs at the other side for detection in any basis 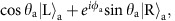 with superposition parameters *θ*_a_, *φ*_a_. In our experiment, *θ*_s_ can be controlled by adjusting the amplitudes of the two frequency components (corresponding to paths L and R, respectively) in the radio-frequency (RF) signal sent into the signal AODs, and *φ*_s_ can be controlled by adjusting the phase of the RF signal component corresponding to the path R. The parameters *θ*_a_ and *φ*_a_ are controlled through the same method by adjusting the amplitudes and phases of the RF signals sent into the idler AODs. By choosing the appropriate parameters *θ*_s_,*φ*_s_ and *θ*_a_,*φ*_a_, we can perform quantum state tomography of the underlying entangled state generated in experiments, and the results are shown in Fig. 3 for different pairs of the memory cells from the center of the ensemble to the edge. The entanglement fidelity *F*_e_, defined as the overlap of the reconstructed experimental density operator with a maximally entangled two-qubit state, is shown in Fig. 3a. All the detected pairs give a high entanglement fidelity, far above the criterion of *F*_e_>1/2 for claim of entanglement.

**Figure 4:**
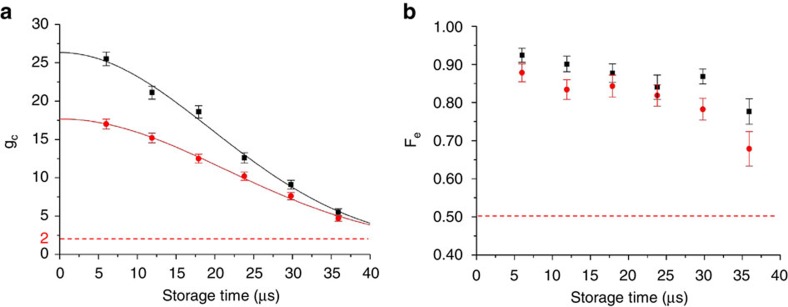
Measurement of quantum storage time in the multi-cell quantum memory. (**a**) Decay of the cross correlation *g*_c_ between the signal and the idler photons with the storage time for a memory cell at the center (squares) with the cell index (8, 8) and at the edge (circles) with the cell index (15, 8). The solid lines correspond to Gaussian fits *g*_c_=1+*g*_0_ exp(−*t*^2^/*τ*^2^) with *g*_0_=25.3 (16.7) and *τ*=27.5 *μs* (30.1 *μs*) for the upper (lower) curves. The dashed line with *g*_c_=2 corresponds to the limit above which we have nonclassical correlation. (**b**) Decay of the entanglement fidelity *F*_e_ with the storage time for entanglement between a pair of memory cells at the center (squares) and the edge (circles) of the 2D array and the flying signal photon. The dashed line with *F*_e_=1/2 corresponds to the limit above which we have genuine quantum storage of entanglement impossible to be emulated by classical operations. Error bars in (**a**,**b**) denote one s.d.

To characterize the storage time of the quantum memory, in Fig. 4 we show decay of the cross correlation *g*_c_ and the entanglement fidelity *F*_e_ for typically memory cells. The fits to the data for *g*_c_ give a *e*^−1^ decay time about 28 μs. The entanglement fidelity *F*_e_ and the cross correlation *g*_c_ are still above the classical limit after 35 μs storage time. The storage time in current experiments is mainly limited by dephasing of the collective mode caused by the motion of atoms and the small residual magnetic field gradient when the magnetic-optical trap is shut off. By loading the atoms into an optical lattice trap, it is possible to extend the storage time to the order of seconds^19^,^20^.

## Discussion

Our experiment realizes a multiplexed quantum memory with hundreds of individually accessible memory cells. The unprecedented large capacity of the DLCZ quantum memory demonstrated in this experiment, together with the programmable control of individual qubits in the memory cells with location independent access time, opens up wide perspective for applications. This type of memory is a critical device required for realization of multiplexed quantum repeater networks for long-distance quantum communication^1^,^2^,^3^,^4^,^5^. With many memory cells, individual and programmable addressing, and efficient interface to the flying optical qubits, this high-capacity quantum memory can also be used for demonstration of many-particle entanglement^31^,^32^ and linear-optics quantum information processing that requires memories^7^,^8^, or as an efficient node in the quantum internet^3^.

## Methods

### Experimental setup

We start by loading ^87^Rb atoms into a magneto-optical trap (MOT) inside a vacuum glass cell. For cooling and trapping of the atoms in the MOT, we use strong cooling beams red detuned to the D2 cycling transition 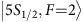
→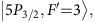 by 12 MHz. The repumping laser, resonant to the 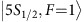
→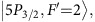 transition, pumps back those atoms which fall out of the cooling cycle. The diameter of the cloud in the MOT is about 3.5 mm and the temperature is about 300 μK. We apply the experimental sequence of the write and read pulses after we shut off the MOT beams and the magnetic gradient coils and wait another 100 μs. For the data reported in Fig. 4 with longer storage time, we also include a polarization gradient cooling (PGC) stage of 1 ms before applying the experimental sequence. The PGC is implemented by increasing the red detuning of the cooling laser to 60 MHz and reducing its intensity to half of the value at the MOT loading stage. The repumping intensity is simultaneously decreased to 0.005 of the value at the loading phase and the magnetic gradient coil is shut off. The temperature is decreased to about 30 μK by the PGC and the size of the MOT remains basically unchanged. After the PGC some portion of the atoms are scattered to the 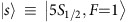 state, and a repumping pulse of 100 μs long is applied after the PGC to pump all the atoms back to the 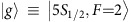 state. The ambient magnetic field is not compensated during the experimental sequence, which induces an oscillation in the retrieval efficiency of the collective atomic excitation by the Larmor frequency^9^,^10^,^11^,^30^. In our experiment, the Larmor period is 5.8 μs and the data in Fig. 4 are taken at integer periods of the Larmor oscillation.

The experimental sequence begins with a write pulse of 100 ns long, red detuned by 10 MHz to the D1 transition 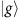
→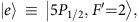 and focused to the atomic ensemble with a 60 μm Gaussian width. The signal photon is collected by a single mode fiber with a focus Gaussian width of 35 μm at the atomic ensemble. If no signal photon is detected, we apply a clean pulse of 100 ns long and resonant to the 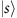
→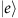 transition, to pump the atoms back to the 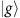 state. The delay between each write pulse and clean pulse is 500 ns, and the write-clean sequence, with a total duration of 1 *μs*, is repeated until a signal photon is detected. When a signal photon is registered, we stop the write-clean sequence. After a controllable storage time, a read laser pulse, same as the clean pulse, is applied to retrieve the collective atomic excitation to an idler photon mode. The conditional control of write/read pulses is implemented by a home-made field-programmable gate array (FPGA). The signal and the idler photons collected by the single-mode fibers are detected by the single-photon counting module, and the events are registered by the coincidence circuit implemented with the FPGA.

### Multiplexing and de-multiplexing optical circuits

Multiplexing and de-multiplexing optical circuits in our experiments are achieved by placing four crossed acoustic-optical deflectors (AODs) in the paths of the write/read beams and the signal/idler photon modes. The crossed AODs, consisting of two AODs in orthogonal directions *X* and *Y*, control the beam deflection angle in these two directions. The crossed AODs, together with the appropriately placed lens, achieve the addressing configuration illustrated in Fig. 1. The AODs are placed on the focal points of the two lens and the two lens are separated by two focal lengths with the atomic ensemble located at the middle point between the two lens. The directions of multiplexing and de-multiplexing AODs are adjusted so that the frequency difference between the signal or idler photon along different paths caused by the multiplexing AODs is canceled after combination by the de-multiplexing AODs, which enables interference between different paths for detection in superposition bases. To compensate the frequency difference induced by different AOD driving frequencies for addressing different cells of the memory array, a double-pass acoustic optical modulator is inserted in the write and the read laser beams to fix their detuning at the atomic cells. As different optical paths in this multiplexing and de-multiplexing circuits go through the same apparatus, the relative phase differences between them are intrinsically stable and we do not need active phase stabilization between different paths.

In our experiment, the mode-matching condition is fulfilled by counter-propagation of the write and read beams and also the signal and idler modes. To adjust matching of the spatial modes, we require the laser beam emitted from the write (signal) fiber can be coupled into the read (idler) fiber at the other side after transmission through all the optical elements, no matter which atomic cells of the memory array they point to. In our experiment, we achieve over 70% coupling efficiency for all the 225 optical paths addressing different memory cells.

The radio frequency (RF) signals to control the AODs are generated by two four-channel arbitrary-waveform generators (AWG). The four analog channels of each AWG output RF with the same frequency and a controllable phase. The four channels in one AWG control the AODs for the write beam, the read beam, the signal mode and the idler mode, respectively, all addressed to the same atomic memory cell. The RF with a different frequency generated by another AWG then applies to these AODs to direct the beams to a different memory cell. We combine the RFs of different frequencies from these two AWGs into a single output for each of the four channels using a RF combiner. These combined outputs are amplified to supply the driving field for the four crossed AODs. The phase at each AOD can be controlled with a precession about 0.1°. To address different memory cells, the frequency of the RF for the crossed AODs is scanned from 98.1 MHz to 107.9 MHz in step of 0.7 MHz in both *X* and *Y* directions, which deflect the beams to 225 different optical paths pointing to the 15 × 15 atomic memory cells.

To align the setup and fulfill the phase matching condition, we use the following alignment procedure: (1) Set the RF frequency in all the 4 pairs of crossed AODs to aim at the middle cell of the 2D scanning array. (2) Send a resonant probe laser 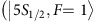
→
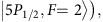
 into the idler fiber and this beam counter-propagates with the idler photon to be scattered by the atoms, then steer the beam to achieve the highest optical depth. (3) Send another resonant pumping beam 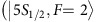
→
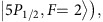
 from the write fiber to overlap with the probe laser aligned in step 2 to achieve the best electromagnetically induced transparency (EIT) signal of the probe laser. (4) Couple the laser from the idler fiber to the signal fiber, and couple the laser from the write fiber to the read fiber. (5) Optimize the scanning ability by fine tuning of the idler AODs, lens 1, atomic ensemble, lens 2 and the signal AODs to form a 4f system (see Fig. 1 in the main text). Do the same tuning to the write AODs, lens 1, atomic ensemble, lens 2 and the read AODs.

We use quantum state tomography to characterize the generated entanglement. The quantum state tomography follows the standard procedure as described in^33^. We reconstruct the density matrix of the entangled state of the atomic memory cells and the signal photon by the maximum likelihood method, based on measurements in 4^2^ bases. The measurement bases we used here are 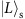, 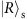, 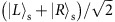, 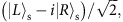 for the signal photon, and 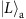, 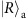, 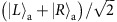, 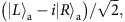 for the atomic memory cells.

### Data availability

The data that support the findings of this study are available from the corresponding author on request.

## Additional information

**How to cite this article:** Pu, Y.-F. *et al*. Experimental realization of a multiplexed quantum memory with 225 individually accessible memory cells. *Nat. Commun.* **8,** 15359 doi: 10.1038/ncomms15359 (2017).

**Publisher's note**: Springer Nature remains neutral with regard to jurisdictional claims in published maps and institutional affiliations.
